# Prognostic Role of Pretreatment C-Reactive Protein to Albumin Ratio in Urological Cancers: A Systematic Review and Meta-Analysis

**DOI:** 10.3389/fonc.2022.879803

**Published:** 2022-04-11

**Authors:** Minhong Wu, Yan Zhou, Qingsheng Chen, Zhiling Yu, Hongyong Gu, Pengxiu Lin, Yanling Li, Cailing Liu

**Affiliations:** ^1^ Department of Urology, Yichun People’s Hospital, Yichun, China; ^2^ Department of Nursing, Wanzai County Traditional Chinese Medicine Hospital, Yichun, China

**Keywords:** urological cancer, C-reactive protein, albumin, prognosis, meta-analysis

## Abstract

**Background:**

To investigate the potential prognostic role of C-reactive protein to albumin ratio (CAR) in patients with urinary cancers, including renal cell carcinoma (RCC), bladder cancer (BC), and prostate cancer (PC).

**Methods:**

We searched and screened literatures with PubMed, Embase, Cochrane Library, and Web of Science in January 2022. We applied combined hazard ratios (HRs) and 95% confidence intervals (CIs) to assess the associations.

**Results:**

Thirteen studies including 2,941 cases were analyzed in our study. Merged results indicated that highly pretreated CAR was associated with inferior overall survival (HR 2.21, 95% CI 1.86-2.62, p < 0.001) and progression-free survival (HR 1.85, 95% CI 1.36-2.52, p < 0.001) for urinary cancers. In a subgroup analysis of OS by tumor type, CAR can be a predictor in RCC (HR 2.10, 95% CI 1.72-2.56), BC (HR 3.35, 95% CI 1.94-5.80), and PC (HR 2.20, 95% CI 1.43-3.37). In a subgroup analysis of PFS by tumor type, CAR can be a predictor in BC (HR 1.76, 95% CI 1.03-3.02), and RCC (HR 1.90, 95% CI 1.25-2.89). The reliability and robustness of results were confirmed.

**Conclusions:**

High pretreated CAR was effective predictor of poor survival in patients with urinary cancers and can act as prognostic factor for these cases.

**Systematic Review Registration:**

PROSPERO (CRD42022306414).

## Introduction

Urinary neoplasms, including renal cell carcinoma (RCC), bladder cancer (BC) and prostate cancer (PC), are usual cancers with increased morbidity and mortality. These three tumors were among the 10 most common malignancies in the United States in 2019 (1). In general, most cancers of the urinary system are found at a local stage. Tumor excision is the preferred management and can achieve well-pleasing outcomes. Nevertheless, some patients may develop metastasis at initial diagnosis, and most local tumors eventually progress to relapsing or metastatic disease. With the development of molecular targeted drugs (2, 3) and immunotherapy (4), the survival of urinary tumors has been greatly improved. However, the long-term survival of these tumors remains disappointing. Therefore, it is of interest to study the prognostic biomarkers in these cases in order to better understand their underlying mechanisms and contribute to the optimal treatment of urinary tumors.

There was increasing evidence of a link between tumor-caused inflammatory response and tumorigenesis and disease progression (5). Kinds of inflammatory and immune response factors have been reported as survival biomarkers for various cancers (6). The C-reactive protein to albumin ratio (CAR) is a measure of serum C-reactive protein (CRP) and albumin and is generated as the CRP level divided by the albumin level. The ratio was originally suggested to forecast the prognosis of acute hospitalized patients (7). Recently, various centers have explored the application of CAR as a prognostic biomarker for kinds of cancers, including gastric cancer (8), colorectal cancer (9), non-small-cell lung cancer (10), gallbladder cancer (11), etc. As for urinary neoplasms, a lot of studies have examined the prognostic value of CAR in RCC. After combining data from these studies, a meta-analysis confirmed that high pretreated CAR was effective predictor of poor survival in cases with RCC (12). Nevertheless, data with potential bias from univariable analysis was included, and the latest studies could not be included (13). Moreover, several studies focusing on the prognostic role of CAR in BC and PC have been published (14–17). This situation encouraged us to conduct the present study to present an integrated review of all related evidence exploring the value of CAR on outcomes in urological cancer patients.

## Methods

### Literature Searching

Our study was performed and presented following the PRISMA guidelines. The protocol has been presented on PROSPERO (No. CRD42022306414). A comprehensive literature search was conducted with MEDLINE, Embase, Cochrane Library, and Web of Science electronic databases. We searched and screened the potential literatures up to January 2022. The key words embraced: “C-reactive protein to albumin ratio” (e.g., “CAR”, “C-reactive protein to albumin ratio”, “C-reactive protein-to-albumin ratio”, “C-reactive protein/albumin ratio”), “urinary cancer” (e.g., “renal cell carcinoma”, “bladder cancer”, “prostate cancer”, “urothelial carcinoma”, “testicular cancer”, “penile cancer”) and “prognosis” (e.g., “survival”, “prognosis”, “outcome”, “progression”, “recurrence”, “metastasis”, “mortality”). A manual screen of study references was also conducted to obtain possibly relevant literatures. The language of included studies was restricted to English.

### Study Inclusion and Exclusion

To be eligible for inclusion, studies must meet the following criteria: (1) studies focusing on the association between pretreated CAR and prognosis of urological cancers, including renal cell carcinoma (RCC), bladder cancer (BC), prostate cancer (PC); (2) studies reported the correlation of pretreated CAR with overall survival (OS), and progression-free survival (PFS); (3) studies directly offered hazard ratios (HRs) with 95% confidence intervals (CIs) in the multivariable cox analyses; (4) studies published in peer-reviewed journals. Exclusion studies according to exclusion criteria: (1) studies didn’t provided HRs with 95% CIs for pretreated CAR in the multivariable cox analyses; (2) duplicated studies; (3) not original articles, such as conference abstracts, reviews, letters or case reports. Two researchers (QSC, ZLY) screened and assessed the literatures independently. The dispute was settled through discussion.

### Data Extraction

In the light of a pre-designed standardized form, data were extracted by two researchers (QSC, ZLY) as following: study features (author’s name, year of publication, region, study design, and number of study cases); patient and tumor characteristics (age, cancer type [renal cell carcinoma, bladder cancer, prostate cancer], cancer stage, detailed treatment strategy, follow-up duration, endpoints of outcome); characteristics of study methodology (detailed value of cut-off, determine method of cut-off, statistical methods of cox analyses, adjusted factors); the detailed HRs with 95% CIs of each study and endpoint.

### Quality Evaluation

The New-castle-Ottawa Scale (NOS) was applied to assess the quality of the included studies. There were 9 items in total (18). The value of NOS higher than 6 was believed as high quality.

### Statistical Analysis

HRs with related 95% CI in each qualified study were combined to assess the prognostic role of pretreated CAR in cases with urinary tumors. In each meta-analysis, Higgins I-Squared statistics and Cochran’s Q test were used to evaluate heterogeneity among the enrolled literature. I^2^ > 50% and/or P<0.1 were believed as measures of significant heterogeneity. In the case of significant heterogeneity, the random-effects model was applied to calculate the aggregate HRs and 95% CI. Otherwise, the fixed effects model was applied. Subgroup analyses of overall survival and progression-free survival were also conducted. Publication bias was evaluated by funnel plots, checked by Egger’s and Begg’s tests. We also conducted sensitivity analysis to examine the stability of the findings. Stata 12.0 (Stata Corporation, College Station) was applied for all statistical analyses.

## Results

### Included Literature

**Figure 1:**
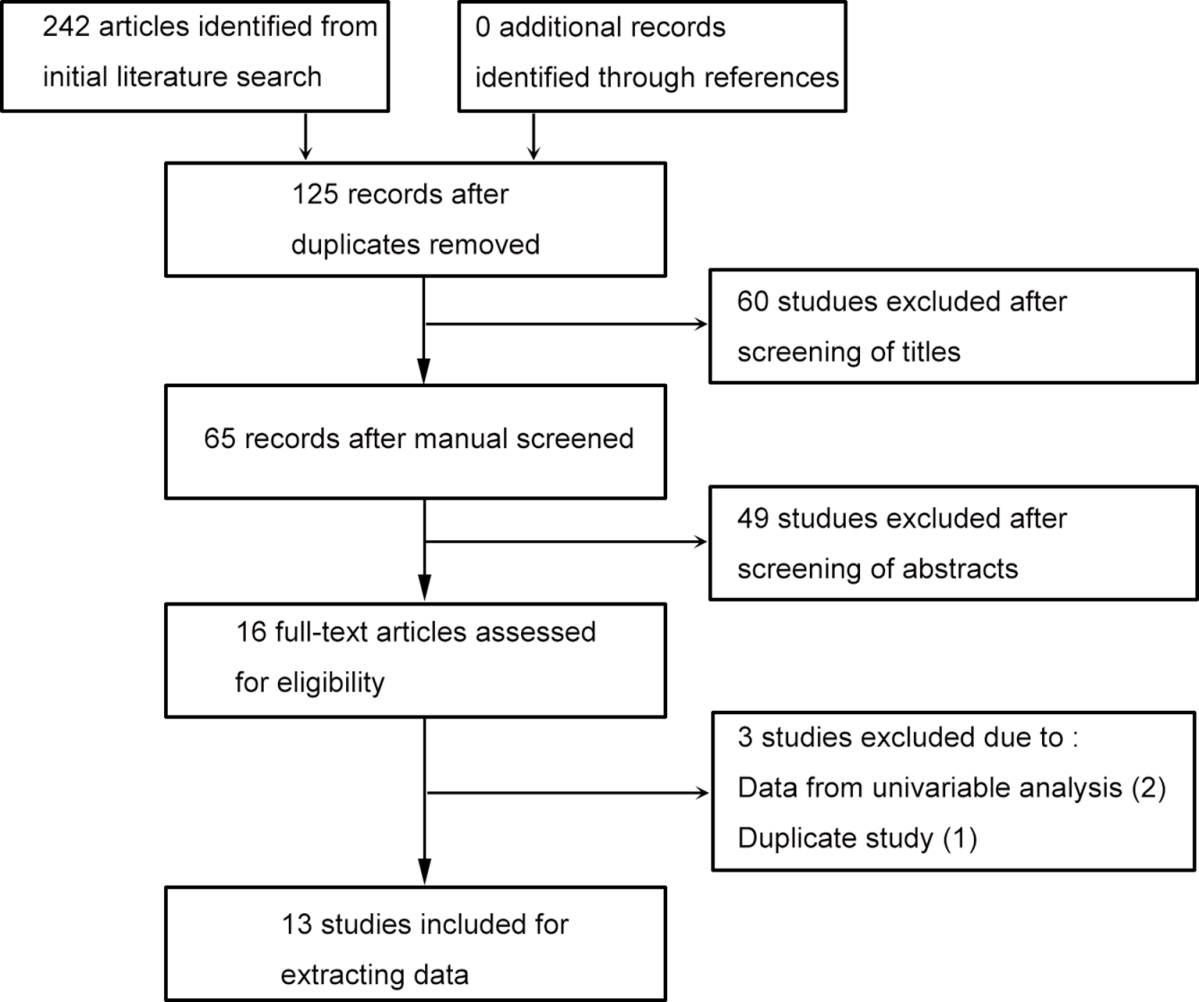
Flowchart of study searching and screening.

Literature search identified 242 studies, and no article was discovered through reference screening. As shown in the flow chart of the literature searching (**Figure 1**), 125 records retained after excluding duplicated studies. After screening literature titles and abstracts, 16 full-text papers retained for next evaluation. Two papers were removed due to lacking data from multivariable analysis, one article was excluded due to duplicate study. Finally, 13 literatures were included for evidence synthesis (13–17, 19–26).

### Study Features

Only data on RCC, BC and PC was identified, studies about the prognostic role of CAR in other urinary cancers were lacking. **Table 1** outlined the main features of all enrolled studies. All studies were published recently (2015–2021) by Asian (Chinese, Japanese and India) researchers. All studies retrospectively analyzed data, nine of them based on single-center patients, four of them based on multi-institutional patients. The median or mean age of subjects ranged from 37 to 73.5 years, and the sample size ranged from 31 to 699. Of all studies, three looked at BC (14–16), eight looked at RCC (13, 19–24, 26), and two looked at PC (17, 25). Most patients were in various stages of the disease and undergo surgery. All studies provided the results of a multivariate Cox analysis. The adjusted factors included patient and tumor characteristics, as detailed in **Table 2**. All studies had high quality, with NOS scores ranging from 6 to 8, as shown in **Table 3**.

**Table 1:** Baseline features of included studies.

Author	Year	Country	Study Design	Case Number	Age (Years)	Cancer type	Stage	Treatment	Cut-off (g/L)	Determine the cut-off value	COX
Zhang (14)	2021	China	RTP, SC	127	66 (29–87) R	BC	Non-metastatic	Surgery	0.165	ROC curve	MV
Kuroda (15)	2021	Japan	RTP, SC	102	71 (49-83) R	BC	Non-metastatic	Surgery	0.17	ROC curve	MV
Ueda (13)	2020	Japan	RTP, SC	131	67 (21-91) R	RCC	All	Targeted therapy	1	Median	MV
Zhang (16)	2019	China	RTP, SC	209	67 (29-87) R	BC	Non-metastatic	Surgery	0.2	X-tile	MV
Uchimoto (17)	2019	Japan	RTP, MI	221	73.5 ± 7.6	PC	All	Androgen-signaling inhibitors or docetaxel	0.5	ROC curve	MV
Tsujino (19)	2019	Japan	RTP, MI	699	61.9 ± 11.7	RCC	All	Surgery	0.073	ROC curve	MV
Konishi (20)	2019	Japan	RTP, MI	176	67 (59-74)	RCC	Metastatic	Targeted therapy	0.05	ROC curve	MV
Gao (21)	2019	China	RTP, SC	108	57 (23-78) R	RCC	All	Surgery	0.094	ROC curve	MV
Barua (22)	2019	India	RTP, SC	31	62 ± 3.14	RCC	Metastatic	Surgery	0.11	ROC curve	MV
Agizamhan (23)	2018	China	RTP, MI	82	37 (2-71) R	RCC	All	Surgery	0.083	ROC curve	MV
Guo (24)	2017	China	RTP, SC	570	51.43 ± 13.52	RCC	All	Surgery	0.08	ROC curve	MV
Yamashita (25)	2016	Japan	RTP, SC	79	72 (52-86) R	PC	All	Chemotherapy	0.07	NR	MV
Chen (26)	2015	China	RTP, SC	406	58 (24–80) R	RCC	All	Surgery	0.06	ROC curve	MV

RTP, retrospective; SC, single center; MI, multi-institutional; BC, bladder cancer; RCC, renal cell carcinoma; PC, prostate cancer; ROC, receiver-operating characteristic; MV, multivariate; NR, not reported; R, range.

**Table 2:** Follow-up and oncological outcomes.

Author	Year	Follow-up duration, month	Outcome	Adjusted factors
Zhang (14)	2021	NR	PFS	Smoking, tumor size, T stage, N stage, AGR, NLR, PLR, albumin, hemoglobin
Kuroda (15)	2021	38.9 (6.1-162.2) R	PFS	Pathological T stage, tumor grade, lymph node metastasis, lymphovascular invasion, preoperative eGFR, postoperative CAR, preoperative PLR
Ueda (13)	2020	NR	OS, PFS	Performance status, prior nephrectomy, IMDC risk classification, albumin, CRP, NLR, NLR/albumin ratio
Zhang (16)	2019	NR	OS	Systemic immune inflammation index, T stage, N stage, M stage, tumor size, tumor margin, vessel invasion, NLR, PLR
Uchimoto (17)	2019	14	OS	ADT duration, visceral mets at first-line treatment, bone mets at first-line treatment, ECOG-PS at first-line treatment
Tsujino (19)	2019	73	OS, PFS	ECOG-PS, T classification, metastasis at diagnosis, UISS, BMI, tumor size, nuclear grade, NLR
Konishi (20)	2019	NR	OS	Age, ECOG PS, sex, IMDC model
Gao (21)	2019	54.5 (7.3-74.2) R	OS	Subtype, fuhrman grade, T stage, N stage, M stage, platelet level
Barua (22)	2019	16.5 ± 1.5	OS, PFS	Age, T stage, fuhrman nuclear grade, tumor necrosis, lymph node status, microscopic invasion, PLR, LMR, systemic immune inflammation index, scan to surgery time
Agizamhan (23)	2018	31 (2–108) R	OS, PFS	Age, fuhrman grade, pT status, pN status, tumor thrombus, NLR, PLR
Guo (24)	2017	NR	OS, PFS	Age, BMI, pathological type, fuhrman grade, pT status, pN status, pM status, serum globulin, NLR, PLR
Yamashita (25)	2016	15.1 (1.8-53.4) R	OS	Age, ECOG PS, significant pain, combination therapy, PSA at docetaxel initiation, androgen deprivation therapy administration period, hemoglobin, NLR, ALP, LDH
Chen (26)	2015	63 (1-151) R	OS	Age, TNM stage, tumor necrosis, lymphovascular invasion, hemoglobin, Ca, GPS, mGPS

PFS, progression-free survival; OS, overall survival; AGR, albumin/globulin ratio; NLR, neutrophil to lymphocyte ratio; PLR, platelet to lymphocyte ratio; eGFR, estimated glomerular filtration rate; CAR, C−reactive protein to albumin ratio; IMDC, International Metastatic Renal Cell Carcinoma Database Consortium; CRP, C-reactive protein; ADT, androgen deprivation therapy; UISS, UCLA integrated staging system; BMI, body mass index; ALP, alkaline phosphatase; LDH, lactate dehydrogenase; GPS, the Glasgow Prognostic Score; NR, not reported; R, range.

**Table 3:** Newcastle–Ottawa scale for risk of bias assessment.

Study	Year	Selection				Comparability	Outcome			Overall
		Representativeness of exposed cohort	Selection of nonexposed	Ascertainment of exposure	Outcome not present at start		Assessment of outcome	Adequate follow-up length	Adequacy of follow-up	
Zhang (14)	2021	1	1	1	1	1	1	1	0	7
Kuroda (15)	2021	1	1	1	1	0	1	1	1	7
Ueda (13)	2020	1	1	1	1	0	1	1	0	6
Zhang (16)	2019	1	1	1	1	1	1	1	0	7
Uchimoto (17)	2019	1	1	1	1	1	1	1	1	8
Tsujino (19)	2019	1	1	1	1	1	1	1	1	8
Konishi (20)	2019	1	1	1	1	1	1	1	0	7
Gao (21)	2019	1	1	1	1	1	1	1	1	8
Barua (22)	2019	0	1	1	1	0	1	1	1	6
Agizamhan (23)	2018	1	1	1	1	0	1	1	1	7
Guo (24)	2017	1	1	1	1	0	1	1	0	6
Yamashita (25)	2016	0	1	1	1	0	1	1	1	6
Chen (26)	2015	1	1	1	1	0	1	1	1	7

### CAR and OS in Urological Cancers

**Figure 2:**
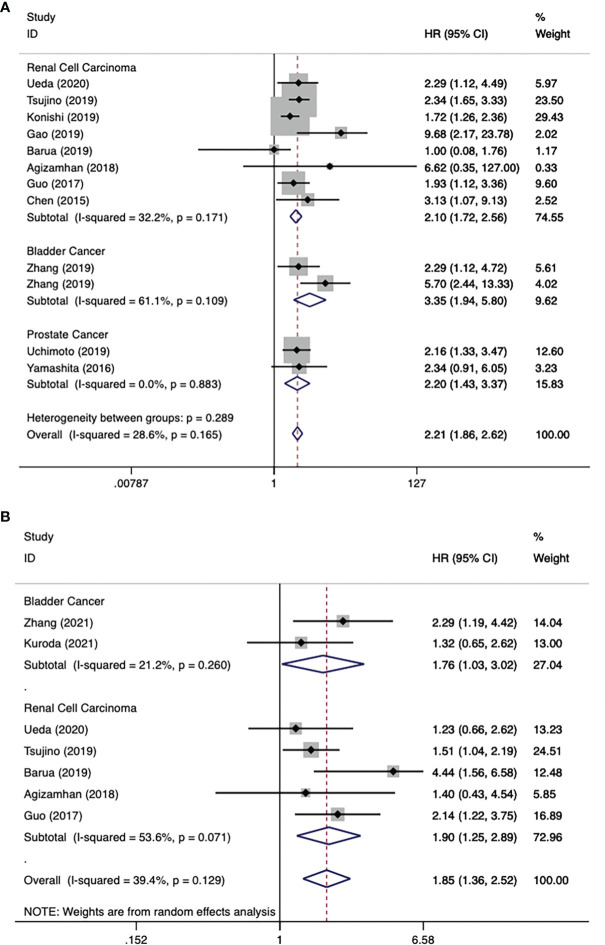
Forest plot reflects the association between CAR and OS/PFS for urological cancers. **(A)** CAR and OS; **(B)** CAR and PFS.

There were 11 literatures on OS of urological cancers (13, 16, 17, 19–26). Since there was no significant heterogeneity among these literatures (I^2^ = 28.6%, p = 0.165), a fixed model was used for analysis. Pooled results showed that higher levels of CAR were associated with poorer OS for urological cancers (HR 2.21, 95% CI 1.86-2.62, p < 0.001). Subgroup analysis by cancer type showed that higher CAR levels were associated with poorer OS in RCC (HR 2.10, 95% CI 1.72-2.56, p < 0.001), BC (HR 3.35, 95% CI 1.94-5.80, p < 0.001), and PC (HR 2.20, 95% CI 1.43-3.37, p < 0.001) (**Figure 2A**). In addition to cancer type, a subgroup analysis of OS included many other variables, including year of publication, region, study design, sample size, cancer stage, and so on. The results for these grouping variables were still significant (**Table 4**).

**Table 4:** Subgroup analysis of overall survival and progression-free survival.

Subgroup	Studies	HR (95% CI)	P value	Heterogeneity	
				I^2^ (%)	P value
*Overall survival*					
Year of publication					
2019-2020	8	2.44 (1.79-3.31)	<0.001	50.8	0.047
2015-2018	4	2.23 (1.45-3.43)	<0.001	0.0	0.760
Region					
China	6	2.95 (2.09-4.17)	<0.001	45.2	0.104
Others	6	2.02 (1.66-2.45)	<0.001	0.0	0.735
Study design					
Single center	8	2.64 (1.97-3.53)	<0.001	35.4	0.146
Multi-institutional	4	2.02 (1.64-2.49)	<0.001	0.0	0.492
Sample size					
> 200	6	2.40 (1.92-3.00)	<0.001	0.50	0.413
< 200	6	1.98 (1.52-2.57)	<0.001	45.7	0.101
Site of carcinoma					
Renal Cell Carcinoma	8	2.10 (1.72-2.56)	<0.001	32.2	0.171
Bladder Cancer	2	3.51 (1.44-8.55)	0.006	61.1	0.109
Prostate Cancer	2	2.20 (1.43-3.37)	<0.001	0.0	0.883
Cancer stage					
All	8	2.38 (1.91-2.96)	<0.001	0.0	0.459
Non-metastatic	2	3.51 (1.44-8.55)	0.006	61.1	0.109
Metastatic	2	1.68 (1.24-2.29)	0.001	0.0	0.509
Cut-off value					
> 0.1	5	2.45 (1.79-3.35)	<0.001	25.6	0.251
< 0.1	7	2.12 (1.73-2.59)	<0.001	36.5	0.150
NOS score					
>= 7	8	2.62 (1.90-3.62)	<0.001	50.4	0.049
< 7	4	2.02 (1.38-2.96)	<0.001	0.0	0.798
*Progression-free survival*					
Year of publication					
2020-2021	3	1.57 (1.06-2.33)	0.023	0.0	0.371
2017-2019	4	2.12 (1.29-3.46)	0.003	58.7	0.064
Region					
China	3	2.09 (1.40-3.12)	<0.001	0.0	0.771
Others	4	1.77 (1.07-2.95)	0.027	65	0.035
Study design					
Single center	5	2.04 (1.33-3.11)	0.001	51.1	0.085
Multi-institutional	2	1.50 (1.05-2.14)	0.026	0.0	0.912
Sample size					
> 130	3	1.59 (1.20-2.11)	0.001	0.0	0.433
< 130	4	2.23 (1.53-3.25)	<0.001	52.2	0.099
Site of carcinoma					
Renal Cell Carcinoma	5	1.90 (1.25-2.89)	0.003	53.6	0.071
Bladder Cancer	2	1.77 (1.10-2.86)	0.019	21.2	0.260
Cut-off value					
> 0.1	4	2.01 (1.14-3.55)	0.016	63.2	0.043
< 0.1	3	1.66 (1.23-2.24)	0.001	0.0	0.572
NOS score					
>= 7	4	1.59 (1.20-2.11)	0.001	0.0	0.662
< 7	3	2.25 (1.15-4.42)	0.018	68.7	0.041

HR, hazard ratio; CI, confidence interval; NOS, Newcastle-Ottawa Scale.

### CAR and PFS in Urological Cancers

There were 7 literatures on PFS of urological cancers (13–15, 19, 22–24). Pooled results showed that higher levels of CAR were associated with poorer PFS for urological cancers (HR 1.85, 95% CI 1.36-2.52, p < 0.001). Subgroup analysis by cancer type showed that higher CAR levels were associated with poorer PFS in BC (HR 1.76, 95% CI 1.03-3.02, p = 0.039), RCC (HR 1.90, 95% CI 1.25-2.89, p = 0.003) (**Figure 2B**). In addition to cancer type, a subgroup analysis of PFS included many other variables, including year of publication, region, study design, sample size, and so on. The results for these grouping variables were still significant (**Table 4**).

### Publication Bias

**Figure 3:**
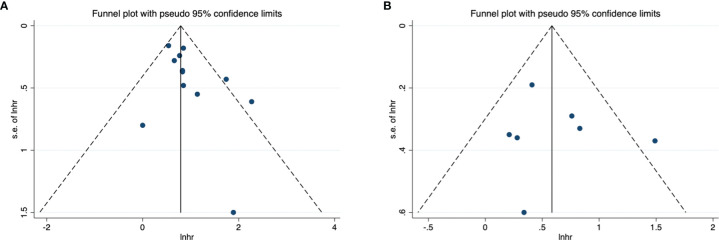
Funnel plot for publication bias. **(A)** correlation of CAR with OS in urological cancers; **(B)** correlation of CAR with PFS in urological cancers.

The funnel diagram of OS and PFS was shown in **Figure 3**, and the two were visually symmetric. Begg’s and Egger’s quantitative tests also showed that there was a low probability of publication bias for OS (P = 0.104 and 0.182) and PFS (P = 0.368 and 0.659).

### Sensitivity Analysis

**Figure 4:**
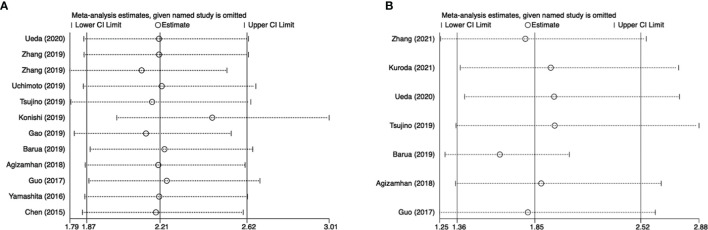
Results of sensitivity analysis. **(A)** correlation of CAR with OS in urological cancers; **(B)** correlation of CAR with PFS in urological cancers.

Sensitivity analysis was further conducted to examine the effect of a single study on the overall results. There was no significant change in HRs associated with CAR and OS or PFS in patients with urinary cancers (**Figure 4**).

## Discussion

The present study attempted to systematically review the available literatures and to evaluate the prognostic significance of CAR in urogenital malignancies using a meta-analysis. Based on high-quality studies, high level of pretreated CAR was found to be significantly correlated to poorer OS and PFS after combining the data. Subgroup analysis by cancer type remained significant for RCC, BC, and PC. Subgroup analyses of several variables of OS and PFS did not alter the direction of the findings. Publication bias checks and sensitivity analyses also confirmed the reliability and robustness of our findings. CRP and albumin are common hematologic indexes in clinic, which are easy to measure and low cost. Thus, CAR can be applied as a competent prognostic indicator for urinary tumors.

For urological tumors, a comprehensive meta-analysis initially examined CAR as a prognostic factor in cases with RCC. They identify that high pretreated CAR was correlated to poorer OS and PFS (12). The associations of CAR and clinicopathological characteristics were also explored. High level of pretreatment CAR was identified to associated with several adverse factors, such as higher Fuhrman grade, higher TNM stage, venous thrombus formation, lymph node invasion, distant metastasis. However, the data from univariable analysis was included, which may bring in potential bias (27). And newly published articles cannot be included. Moreover, several studies investigating the prognostic role of CAR in BC and PC have been published (14–17). In this case, we only included data from multivariable analyses. The present study provided the latest and most integrated evidence of the prognostic role of CAR in urinary tumors. Based on adjusted data, we have found similar results to study by Zhou et al. (12). More detailed results about BC and PC were reported in the present study. Pretreatment CAR remained to be an important prognosis predictor for patients with BC and PC.

Inflammatory reactions promote the development of tumors by influencing the microenvironment of urinary tumors. Cancer cell proliferation, necrosis, invasion, and hypoxia trigger immune responses in the tumor microenvironment, and in turn trigger the generation of various of inflammatory factors (28). Serum C-reactive protein and albumin are indexes of chronic inflammation and malnutrition in cancer patients (29, 30). C-reactive protein is an acute phase protein generated in the liver that stimulates cancer-related inflammatory factors such as IL-1, IL-6, and TNF-A, resulting in progression of malignance (31). The studies showed that high CRP level was associated with poorer survival outcomes in urological cancer cases (32). Serum albumin level reflects the nutritional status of patients. Low serum albumin levels indicate malnutrition. Hypoalbuminemia is caused by nutrient intakes and tumor overconsumption and induces stimulation of inflammatory factors such as IL-1, IL-6, and TNF-A (33). CAR was generated in the light of CRP and albumin levels. Thus, CAR provides a biological basis and is considered a promising prognostic tool for urinary tumors.

There was increasing evidence of a link between tumor-caused inflammatory response and tumorigenesis and disease progression (5). Kinds of inflammatory and immune response factors have been reported as survival biomarkers for various cancers (6). Many other inflammatory biomarkers that were also prognostic in urologic tumors, such as neutrophil-to-lymphocyte ratio (NLR) (34), platelet-to-lymphocyte ratio (PLR) (35), lymphocyte-to-monocyte ratio (LMR) (36), etc. Based on published studies, similar meta-analyses about the prognostic role of these biomarkers in urological tumors also have been performed by many researchers. They are common hematologic indexes in clinic, easily to measure and low cost, which can be widely used. However, which marker was the best predictor, the studies comparing these markers were inadequate. We believed that combining these markers for prediction and improving prediction efficiency may be the direction of future researches.

However, some of the limitations of this systematic review should be explained. First of all, only 13 studies have been enrolled in the meta-analysis, which may lack statistical power, especially for BC and PC. Second, all enrolled studies were performed in Asia. Therefore, we should be careful to apply the results of the present study in patients from western countries. Third, all the included studies were cohort studies with retrospective design, which may result in selection bias. Fourthly, different cut-off values of CAR in different studies may lead to inconsistent outcome thresholds. Lastly, due to inadequate literatures, other urinary cancers were not analyzed in our study, and the endpoint cancer-specific survival was not studied.

Taken together, the present study identified that pretreatment CAR level could be a likely predictor for cases with urinary tumors. Nevertheless, well-designed prospective studies also are wanted to validate these results.

## Data Availability Statement

The original contributions presented in the study are included in the article/supplementary material. Further inquiries can be directed to the corresponding author.

## Author Contributions

Protocol/project development: MW and YZ. Data collection or management: QC, ZY, and HG. Data analysis: PL, YL, and CL. Manuscript writing/editing: MW and YZ. All authors read and approved the final manuscript.

## Conflict of Interest

The authors declare that the research was conducted in the absence of any commercial or financial relationships that could be construed as a potential conflict of interest.

## Publisher’s Note

All claims expressed in this article are solely those of the authors and do not necessarily represent those of their affiliated organizations, or those of the publisher, the editors and the reviewers. Any product that may be evaluated in this article, or claim that may be made by its manufacturer, is not guaranteed or endorsed by the publisher.
